# *Dusuqing* granules (DSQ) suppress inflammation in Klebsiella pneumonia rat via NF-κB/MAPK signaling

**DOI:** 10.1186/s12906-017-1736-x

**Published:** 2017-04-17

**Authors:** Xue Mei, Hao-Xun Wang, Jian-Sheng Li, Xiao-Hui Liu, Xiao-Fan Lu, Ya Li, Wei-Yu Zhang, Yan-Ge Tian

**Affiliations:** 1 Basic Medicine College, Henan University of Chinese Medicine, Zhengzhou, Henan 450046 China; 2 Henan Key Laboratory of Chinese Medicine for Respiratory Disease, Henan University of Chinese Medicine, Zhengzhou, Henan 450046 China; 3 Collaborative Innovation Center for Respiratory Disease Diagnosis and Treatment & Chinese Medicine Development of Henan Province, Henan University of Chinese Medicine, Zhengzhou, Henan 450046 China; 4 The Second Affiliated Hospital, Zhengzhou University, Zhengzhou, Henan 450014 China; 5 The First Affiliated Hospital, Henan University of Chinese Medicine, Zhengzhou, Henan 450000 China

**Keywords:** Klebsiella pneumoniae pneumonia, Dusuqing granules, inflammation, NF-κB/MAPK pathway, Traditional Chinese medicine

## Abstract

**Background:**

*Dusuqing* granules (DSQ) have been used in the treatment of bacterial pneumonia clinically, with remarkable benefits. This study was initiated to explore the effects of DSQ on pulmonary inflammation by regulating nuclear factor (NF)-κB/mitogen-activated protein kinase (MAPK) signaling in bacterial pneumonia rats.

**Methods:**

Rat model was duplicated with Klebsiella pneumonia by a one-time intratracheal injection. Rats were randomized into control, model, DSQ and levofloxacin (LVX) groups. After administrated with appropriate medicines for 7 days, lung tissues were harvested and prepared for pathological analysis, and interleukin (IL)-1, IL-6, monocyte chemotactic protein (MCP)-1and macrophage inflammatory protein (MIP)-2 detections. NF-κB mRNA was measured by real-time qPCR, and the phosphorylation and total proteins of P38MAPK, JNK46/54, ERK42/44 were determined by Western blotting.

**Results:**

Marked pathological impairments were observed in model rats, whereas were improved in DSQ group. The cytokines levels, NF-κB mRNA expression and the phosphorylation of P38MAPK, JNK46/54 and ERK42/44 proteins were significantly higher in model group, and were significantly depressed in DSQ group.

**Conclusion:**

The protective effects of DSQ on Klebsiella pneumonia might be attributed to its inactivative effects of NF-κB/ MAPK pathway.

## Background

Klebsiellar pneumonia (KP), an important type of pneumonia typically in the form of bronchopneumonia and also bronchitis, is caused by *Klebsiella pneumoniae* (*K.* pneumoniae)*,* one of the most common Gram-negative pathogens and is becoming increasingly multidrug- resistant. KP is a major public health threat because of its severity, high incidence of complications, and elevated mortality, and its mortality rate was about 50% even under antimicrobial therapy [1–4]. Nuclear factor (NF)-κB/mitogen-activated protein kinase (MAPK) signaling pathway , important pathways involved in inflammation are closely related to the progress of KP. Signals are derived from pathogens or host cells, such as pathogen-associated molecular patterns (PMAP), danger-associated molecular patterns (DAMP). PAMP and DAMP are recognized by various pattern recognition receptors and ultimately cause the activations of MAPK (p38, ERK, JNK) and NF-κB signaling pathways. The pathways can initiate the gene transcriptions and promote expressions of various cytokines, such as interleukin (IL)-1, IL-6, tumor necrosis factor (TNF)-α, monocyte chemotactic protein (MCP)-1, etc., and amplify the inflammatory response through a positive feedback cascade [5, 6]. However, the cytokines production whose contribution to the host may be either protective or detrimental [7, 8].

Pneumonia belongs to the realm of syndrome of wind-heat invading lung in traditional Chinese medicine (TCM). Phlegm-heat syndrome always lasts through the whole process of the disease, and was often combined with qi and yin deficiency. Therefore, the method of heat-clearing, toxin-relieving and phlegm-resolving as well as replenishing qi and yin are the basis treatment of the disease. Based on this theory, our research group drew up the *Dusuqing* granules (DSQ), which has the effects of clearing heat, relieving toxin, resolving phlegm and replenishing qi and yin. In our previous randomized controlled trials, we found that DSQ can alleviate clinical symptoms and signs, improve quality of life of bacterial pneumonia patients [9, 10]. In animal studies, it indicates that DSQ can lessen pulmonary inflammation and impairments, depress the levels of CRP and CR as well as proinflammatory cytokines [11, 12]. In this study, we aimed to explore the effects of DSQ on pulmonary inflammation in bacterial pneumonia rats via regulating NF-κB/MAPK signaling.

## Methods

### Animals

Twenty-eight male and 28 female Sprague-Dawley rats weighing 180 - 220 grams (g) were used in this study. All animals were obtained from Henan Laboratory Animal Center (Zhengzhou, Henan, China) and received humane care. The rats were randomly divided into control, model, DSQ, and levofloxacin (LVX) groups, 14 rats each group. The study protocol was approved by the Experimental Animal Care and Ethics committees in Henan University of Chinese Medicine (Zhengzhou, Henan, China). DSQ (batch: 130727) were purchased from the First Affiliated Hospital, Henan University of Chinese Medicine, Zhengzhou, Henan, China. The main components of the granule are as follows: Ren Shen (Radix ginseng) 10 g, Mai Dong (Ophiopogon japonicus) 15 g, Sheng Di Huang (radix rehmanniae recen)15g, Gua lou(trichosanthes kirilowii Maxim)20g, Yu Xing Cao(Houttuynia cordata)20g, Bai Tou Weng(the root of Chinese pulsatilla)10g. Levofloxacin tablets (batch: 20101001) were purchased from the Daiichi Pharmaceutical (Beijing) co., LTD.

### Klebsiella pneumoniae

*K.* pneumoniae (Strain: 46114, National Center for Medical Culture Collections, Beijing, China) was cultured on nutrient agar plate and diluted to 2.4×10^8^ colony forming unit (CFU)/mL with normal saline before administered to animals.

### Model preparation

KP rat models were prepared according to the reference [13]. After anesthetized with 10% chloral hydrate (2.8 mg/kg) on day 4, *K. pneumoniae* solution (0.1 mL/animal) were given to the model rats with a one-time orotracheal intubation, while normal saline (0.1 ml/animal) were given to the controls.

### Administrations

After adaptive accommodation for 7 days, DSQ (2.8 mg·kg^-1^·d^-1^) and LVX (31.64 mg·kg^-1^·d^-1^) were given to rats in DSQ and LVX groups, respectively, 3 days before KP challenge, while the control and model rats were intragastrically administrated with normal saline (2 ml/animal). The equivalent doses of DSQ and LVX were calculated using the following formula: D_rat_=D_human_ × (K_rat_/K_human_) × (W_rat_/W_human_)^2/3^ [14]. D: dose (mg/kg); K: body shape index; W: body weight. On the 4^th^ day, the models were made. From 5 to 7 day, the gavage solutions were carried out as the first three days. Body weights were measured on day 1 and 5 for the adjustments of the doses. All rats were sacrificed on day 8.

### Cytological analysis in peripheral blood and bronchoalveolar lavage fluid

The population of white blood cells and the percentage of polymorphonuclear neutrophils (PMN) in peripheral blood were measured by Hema-screen 18 (Italy), while they were counted in the bronchoalveolar lavage fluid (BALF) under an inverted microscopes (Olympus, Japan). Blood samples were sampled from caudal vein before sacrificed, and the BALFs were prepared with a three-time bronchoalveolar lavaging from the excised left lung lobes.

### Lung water content analysis

Lung wet weights were measured and recorded after the lung lobes were excised, and dry weight were recorded after the lung tissues were totally dried in an air oven. Lung water content (g/100g) = (wet weight - dry weight) × 100/wet weight.

### Morphology assessment

For histological examination, after fixed in 4% paraformaldehyde for 72 hours, the lung tissues were paraffin embedded and sectioned into 4-μm-thick slices, and stained with haematoxylin and eosin (H&E) solutions. Images were captured at amplification of 200 under an Olympus PM-10 AD optical microscope and photographic system (Olympus, Tokyo, Japan). Lung injury, including alveolar congestion, hemorrhage, infiltration or aggregation of neutrophils in airspace or vessel wall, and thickness of alveolar wall, was blinded scored by a pathologist, according to a five-point scales used in previous study [15]: 0 = minimal (little) damage, 1 = mild damage, 2 = moderate damage, 3 = severe damage and 4 = maximal damage. Total lung injury score was calculated as the sum of the above-mentioned four lesions.

### Enzyme linked immunosorbent assay

After the excised lung tissues homogenized in 10× weight normal saline, the supernatant was separated and stored at -80 °C for further analysis. The expressions of IL-1β, IL-6, MCP-1 and MIP-2 in lung tissues were analyzed by enzyme linked immunosorbent assay (ELISA) according the introduction of the kits (Boster, Wuhan, China). The results were represented by pg/ml.

### Quantitative PCR analysis

Total RNA was extracted by using TRIzol reagent (Invitrogen, US) according to the instruction and assessed by agarose gel electrophoresis and absorbance measurements at 260 and 280 nanometer (nm) wavelength on Thermo BIONANO Ultramicro ultraviolet light spectrophotometer (Thermo Forma, MA, USA). Complementary DNA (cDNA) was synthesized with a SuperRT cDNA kit (CW Biotechnology, Beijing, China) according to the manufacturer’s standard protocol, while PCR was performed by using Quantifast® SYBR® Green PCR Kit (QIAGEN, Germany). The reaction systems were prepared following the instructions of the kits. The initial activation was at 95 °C for 15 s, 60 °C for 1 min on an ABI 7500 Fast real time instrument (ABI, CA, USA). The primers of NF-κB and glyceraldehyde-3-phosphate dehydrogenase (GAPDH) were designed and synthesized by Generay Biotech Co. Ltd. (Shanghai, China). NF-κB: forward (5’-3’): ACG ATC TGT TTC CCC TCA TCT, reverse (5’-3’): TGG GTG CGT CTT AGT GGT ATC; GAPDH: forward (5’-3’): GGT GAA GGT CGG TGT GAA CG, reverse (5’-3’): CTC GCT CCT GGA AGA TGG TG.

### Western blotting analysis

Lung tissues were homogenized in ice-cold lysis buffer containing 1 mM phenylmethanesulfonyl fluoride (PMSF) and 1× protease inhibitor cocktail (Sigma-Aldrich, USA) for 40 min, then centrifuged at 12,000 g for 20 min. Protein concentrations were measured using the BCA assay. 30 μg of total protein was sampled and separated in 10% sodium dodecyl sulfate/polyacrylamide gel electrophoresis (SDS-PAGE) and transferred to polyvinylidene difluoride (PVDF) membranes (Millipore, MA, USA). The membranes were then blocked with 5% fat-free milk in Tris-buffered saline and 0.5% Tween-20 (TBS-T) for 2 h and then incubated with corresponding primary antibodies overnight at 4 °C. After washing with TBS-T, the membranes were incubated with horseradish peroxidase (HRP)-conjugated goat anti-rabbit antibody (ZB2306) for 1 h at room temperature. Finally, signals were gained by using the enhanced chemiluminescence (ECL) kit (CW Biotechnology, Beijing, China) and were exposed by using a biological molecular imaging instrument (LAS4000mini, JAPAN). The immunoblots intensities were quantified using Quantity One software (Bio-Rad, CA, USA). The primary antibodies including P38 (D13E1), p-P38 (D3F9), ERK (137F5), P-ERK (D13.14.4E), JNK (56G8), p-JNK (98F2), β-Tublin (9F3) and GAPDH (14C10) were purchased from Cell Signaling Technology, Inc. (CST, MA, USA).

### Statistical analysis

Statistical analyses were performed using SPSS Statistics 19.0 software (SPSS, IL, USA). One-Way ANOVA was applied for comparison among groups. All data were expressed as mean ± standard deviation (SD). *P*<0.05 was set as the statistical significance.

## Results

### Mortality

On day 5 and 6, three rats died in model group and one in LVX group due to pulmonary infection and abscess, one rat died in DSQ group due to flatulence.

### Lung water content

Lung water content of the rats was significantly higher in model group compared with control group (*P*<0.01). Compared with model group, it was significantly lower in DSQ and LVX groups (*P*<0.01). Especially, it was significantly lower in DSQ group than that in LVX group (*P*<0.01) (Table 1).

**Table 1:** Lung Water Content in each group (mean ± SD)

Group	N	Lung water content
control	14	62.30 ± 1.44
model	11	94.06 ± 2.20**
DSQ	13	75.86 ±1.11^##▲▲^
LVX	13	80.80 ± 0.70^##^

Note: ^**^ *P*<0.01 vs. control group; ^##^ *P*<0.01 vs. model group; ^▲▲^ *P*<0.01 vs. LVX group

### Histomorphology

**Fig. 1:**
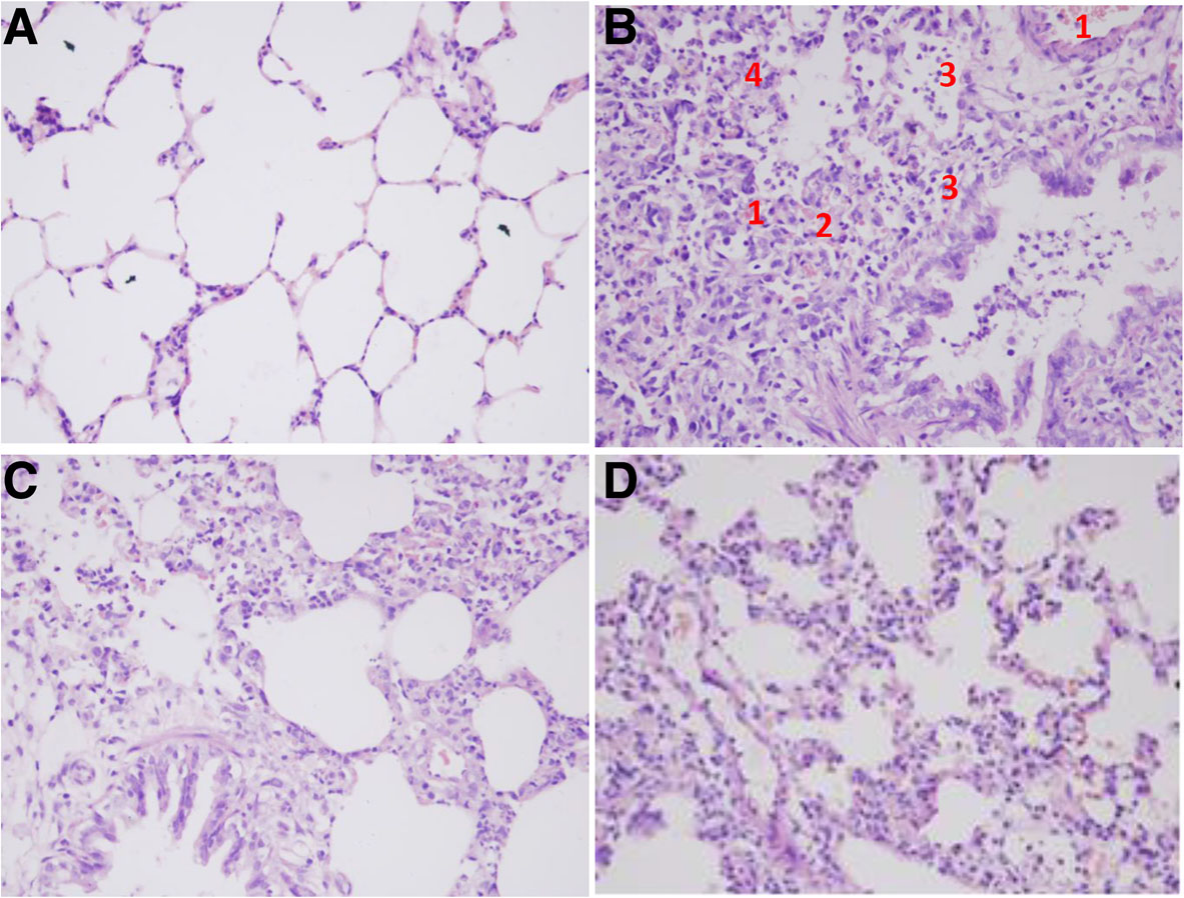
Pulmonary histomorphology in each group (H&E stained). (**a**) control group (**b**) model group (**c**) DSQ group; (**d**) LVX group. 1: alveolar congestion, 2: hemorrhage, 3: infiltration or aggregation of neutrophils, 4: thickness of alveolar wall (B)

As shown in Fig. 1, the lung tissues in model group were found damaged and expressed alveolar congestion, hemorrhage, alveolar wall thickened, and inflammatory cells infiltration in the interstitium and alveolar spaces, and were reduced in DSQ and LVX groups. No apparent impairment was observed in control rats.

Lung injury score was significantly higher in model group compared with controls (*P*<0.01), and it was significantly lower in DSQ and LVX groups (*P*<0.01). There was no significance between DSQ and LVX group (*P* > 0.05) (Table 2).

**Table 2:** Lung Injury Score (mean ± SD)

Group	N	Score
control	14	1.50±0.84
model	11	12.17±0.75**
DSQ	13	8.17±0.75^##^
LVX	13	8.33±0.82^##^

Note: ^**^ *P*<0.01 vs. control group; ^##^ *P*<0.01 vs. model group

### WBC population and PMN percent in peripheral blood

WBC population and PMN percent increased significantly in peripheral blood in model group compared with control group (*P*<0.01), and they were significantly lower in DSQ and LVX groups compared to model rats (*P*<0.01). There was no significance between DSQ and LVX group (*P* > 0.05) (Table 3).

**Table 3:** WBC population and PMN percent in peripheral blood in each group (mean ± SD)

Group	N	WBC (×10^9^/L)	PMN (%)
control	14	6.38±1.23	24.22±5.11
model	11	13.52±2.86**	56.24±6.37**
DSQ	13	8.60±2.86^##^	37.25±4.22^##^
LVX	13	9.29±1.80^##^	35.44±6.42^##^

Note: ^**^ *P*<0.01 vs. control group; ^##^ *P*<0.01 vs. model group

### WBC and PMN populations in BALF

The populations of WBC and PMN in BALF increased significantly in model group compared with control group (*P*<0.01), and were decreased significantly in DSQ and LVX groups (*P*<0.05). There was no significance between DSQ and LVX group (*P* > 0.05) (Table 4).

**Table 4:** WBC and PMN populations in bronchoalveolar lavage fluid in each group (mean ± SD)

Group	N	WBC (×10^6^/L)	PMN (×10^6^/L)
control	14	17.14±2.29	8.82±2.07
model	11	24.45±4.71**	16.64±2.75**
DSQ	13	21.36±3.76^#^	13.65±3.17^#^
LVX	13	20.84±3.34^#^	13.95±3.16^#^

Note: ^**^ *P*<0.01 vs. control group; ^##^ *P*<0.01 vs. model group

### IL-1β, IL-6, MCP-1 and MIP-2 in lung tissue

**Fig. 2:**
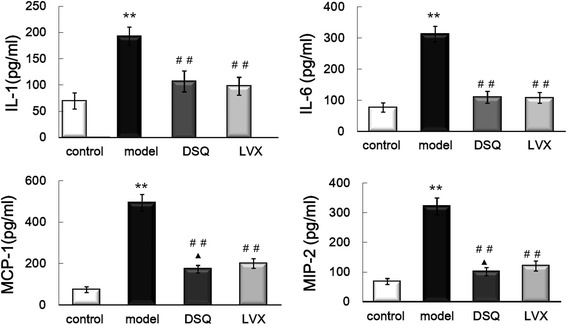
Levels of IL-1β, IL-6, MCP-1 and MIP-2 in the lungs in control, model, DSQ and LVX groups^**^ *P*<0.01 vs. control group; ^##^ *P*<0.01 vs. model group; ^▲^ *P*<0.05 vs. LVX group. Values are expressed as mean ± S.D., one-way ANOVA

As shown in Fig. 2, IL-1β, IL-6, MCP-1 and MIP-2 were significantly higher in model group than control group (*P* < 0.01). Compared with model group, IL-1β, IL-6, MCP-1 and MIP-2 decreased significantly in DSQ and LVX groups (*P*<0.01). MCP-1 and MIP-2 were lower in DSQ group than LVX group (*P* < 0.05).

### Expression of NF-κB mRNA

**Fig. 3:**
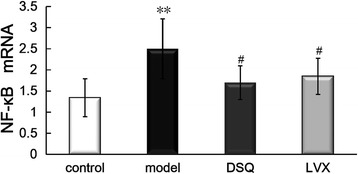
Expressions of NF-κB mRNA in the lungs in control, model, Dusuqing and Levofioxacin^**^ *P*<0.01 vs. control group; ^#^ *P*<0.05 vs. model group;. Values are expressed as mean ± S.D., one-way ANOVA

As shown in Fig. 3, Compared with control group, the expression of NF-κB mRNA in model group increased significantly (*P* < 0.01), while it was significantly lower in DSQ and LVX groups compared to model group (*P* < 0.05).

### P38MAPK, JNK54/46 and ERK1/2 protein and their phosphorylation in each group

**Fig. 4:**
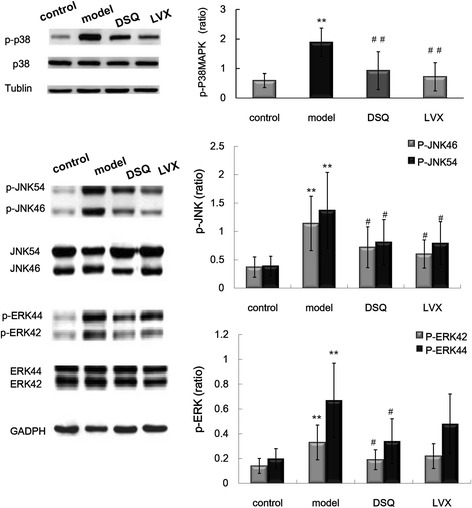
Phosphorylation of P38MAPK, JNK and ERK1/2 in the lungs in control, model, DSQ and LVX groups^**^ *P*<0.01 vs. control group; ^#^ *P*<0.05, ^##^ *P*<0.01 vs. model group. Values are expressed as mean ± S.D., one-way ANOVA

As shown in Fig. 4, Significant elevation of p- P38MAPK , p-JNK and p-ERK1/2 levels was observed in model group compared with control group(*P* < 0.01),while the expressions of p- P38MAPK and p-JNK were significantly lower in DSQ and LVX groups than in model group (*P*<0.05, *P*<0.01). The level of p-ERK1/2 were lower in DSQ group than in model group (*P*<0.05), while there was no difference in p-ERK1/2 level between LVX group and model group (*P*>0.05).

## Discussion

The present study demonstrates that *Dusuqing* granules, an effective formula clinical used for the treatment of community-acquired pneumonia in the elderly play distinctly suppressant roles on inflammatory response in *Klebsiella* pneumonia rats induced by a one-time attack of *K. pneumoniae* infection via regulating NF-κB/ MAPK pathway.

The pattern of phlegm-heat obstructing lungs is one of the most common syndromes in bacterial pneumonia, which happens in the elderly always with deficient vital root, qi-yin deficiency syndrome, or qi-yin impaired by long-term phlegm-heat pathogens. DSQ is specific preparation for the syndromes of phlegm-heat obstructing lungs combined with qi-yin deficiency. It has been confirmed effective in alleviating systemic and pulmonary inflammation and impairments, improving clinical symptoms, and improving quality of life of community-acquired pneumonia patients in previous studies [9–12]. High component complexity of traditional Chinese herbal prescription has its unique advantages and characteristics of therapeutic effects via multi-level, multi-channel and multi-target [16]. In this study, we aimed to reveal the further mechanism of DSQ in treating bacterial pneumonia via the regulation of NF-κB/MAPK signaling.

Klebsiella pneumonia is one kind of most common lung infections. As one of phagocytes that constitute an integral component of innate immune defense, PMNs migrate to inflammation sites to eliminate pathogens, early in the acute inflammatory response to infection and injury [17]. Our data indicated that DSQ can significantly reduce the white blood cell population and PMN rate in peripheral blood. Additionally, DSQ can also alleviate pathological changes in the lungs, including reducing inflammatory cell infiltration in airspace and vessel walls, alleviating pulmonary congestion and hemorrhage, and decreasing alveolar wall thickening. These results are consistent with previous studies [11, 18].

NF-κB is the most important transcriptional regulator that regulate the inflammatory pathways. Activated NF-κB by a wide array of mediators can translocate to the nucleus and bind to the promoters of pro-inflammatory genes, leading to enhanced gene expression and amplification of the inflammatory response which lead to the inflammatory injury. Therefore, blocking NF-κB transcriptional activity may be an important target for treating inflammatory diseases [19–21]. In this study, NF-κB mRNA was observed significantly elevated in the lungs of KP rats, DSQ can suppress the expression of NF-κB mRNA, and DSQ showed similar regulative effects with LVX.

The MAPK signaling pathway, classified into extracellular signal regulated kinases 1/2, c-Jun N-terminal kinase, and p38MAPK, is implicated in the release of immune-related cytotoxic factors and pro-inflammatory cytokines, and inhibitors of MAPK reduce pro-inflammatory protein levels [22, 23]. Data indicate that the activation of NF-κB/MAPK signaling contributes to KP-induced cytokine/chemokine production in the lungs [24]. Decreasing of the phosphorylation level of NF-κB and P38 can down-regulate the productions of cytokines, and minimize the injuries in lung tissue [25]. In this study, we found that the phosphorylation levels of P38 MAPK, JNK and ERK1/2 increased significantly in the lungs of KP rats. DSQ can suppress the phosphorylation of MAPK. Our data indicate DSQ can decrease the productions of IL-1β, IL-6, MCP-1 and MIP-2 which increased significantly in the lungs of KP rats by inhibiting NF-κB and MAPK.

## Conclusion

In conclusion, DSQ have significant effects on reducing pulmonary pathological impairments and inflammatory responses, and the inhibiting effects of NF-κB and MAPK signaling of DSQ might be involved in its underlying mechanisms, and these data provide new evidence for the clinical application of Traditional Chinese Medicine.

## Abbreviations

DSQ: Dusuqing
GAPDH: Glyceraldehyde-3-phosphate dehydrogenase
IL: Interleukin
KP: Klebsiellar pneumonia
LVX: Levofloxacin
MAPK: Mitogen-activated protein kinase
MCP-1: Monocyte chemotactic proteinand-1
MIP-2: Macrophage inflammatory protein-2
NF-κB: Nuclear factor-κB
